# Dietary apple polyphenols increase skeletal muscle capillaries in Wistar rats

**DOI:** 10.14814/phy2.13866

**Published:** 2018-09-17

**Authors:** Yuki Yoshida, Arata Tsutaki, Yuki Tamura, Karina Kouzaki, Koichi Sashihara, Shohei Nakashima, Motoyuki Tagashira, Ryuichi Tatsumi, Koichi Nakazato

**Affiliations:** ^1^ Department of Exercise Physiology Graduate School of Health and Sport Science Nippon Sport Science University Setagaya‐ku Tokyo Japan; ^2^ Asahi Breweries, Ltd., Moriya Ibaraki Japan; ^3^ Department of Animal and Marine Bioresource Sciences Kyushu University Fukuoka Japan

**Keywords:** angiogenesis, apple, polyphenol, thrombosponsin‐1

## Abstract

Dietary apple polyphenols (AP) have been shown to exhibit beneficial effects on muscle endurance. Fast‐to‐slow change in the composition of myosin heavy chains was known as one of the molecular mechanisms. Here, we examined the effects of dietary AP on the capillaries and mitochondria in the rat skeletal muscle to elucidate the mechanisms underlying muscular endurance enhancement. Twenty‐four Wistar male rats were divided into three groups, namely, the control group, 0.5% AP group, and 5% AP group (*n* = 8 in each group). After a feeding period of 4 weeks, rats were dissected, gastrocnemius muscles were removed, and the density of capillaries and levels of mitochondrial proteins were analyzed. Capillary density of the gastrocnemius increased to 17.8% in rats fed with 5% AP as compared to the control rats. No significant change was observed in the mitochondrial content and dynamics (fusion/fission) of regulatory proteins. To investigate the mechanisms underlying the increase in the capillary density, positive (vascular endothelial cell growth factor, VEGF) and negative (thrombosponsin‐1, TSP‐1) factors of angiogenesis were analyzed. TSP‐1 expression significantly decreased in rats fed with 0.5% AP and 5% AP by approximately 25% and 40%, respectively, as compared with the control rats. There were no significant differences in VEGF expression. Thus, dietary AP may increase the muscle capillary density by decreasing TSP‐1 expression. We concluded that the increase in the capillary density and the fast‐to‐slow change in myosin heavy chains by AP feeding are the main causes for muscle endurance enhancement in Wistar rats.

## Introduction

In today's aging society, improvement in physical performances is important for maintaining the quality of life. Exercise intervention is a promising way to enhance both endurance capacity and muscular strength. Several attempts have been made to improve physical performances using oral administration of compounds such as those derived from plants (Ryu et al. 2001; Hodgson et al. 2013; Hsu et al. 2016). The nutraceutical approach is believed to be a promising strategy for individuals who cannot undergo regular training, owing to injuries and diseases.

Polyphenols are ubiquitous in the plant kingdom, and the effects of dietary polyphenols on the muscle endurance have been previously reported. Murase et al. (2005, 2006) reported the effects of the intake of 0.2–0.5% of polyphenol‐rich green tea extract (GTE) for 10 weeks on the endurance capacity in BALB/c mice. These authors demonstrated that the running time to exhaustion was 30% higher in BALB/c mice fed with 0.5% GTE than the control mice, while the swimming time to exhaustion prolonged by 8–24% in the test group than the control mice.

Apple, a member of the Rosaceae family, is the most widely grown fruit crop in the world. Apples are a rich source of polyphenols, and the antioxidant activity of polyphenols from apples is reported to be the strongest among fruits (Eberhardt et al. 2000). Apple polyphenols (AP) comprise a mixture of polyphenols that consist of procyanidine dimers and pentadecamers (about 45% w/w), phenolic acids (about 25% w/w; mainly chlorogenic acid), monomeric flavan‐3‐ols (about 15%; catechin), phloretin glycosides (about 10% w/w; mainly phloridzin), and other components (about 5% w/w; mainly quercetin glycosides) (Kanda et al. 1998; Ohnishi‐Kameyama et al. 1997; Shoji et al. 2000; Hammerstone et al. 2000; Vinson et al. 2001). Procyanidine, the major component, comprises (+)‐catechin and (−)‐epicatechin units that are widely found as secondary metabolites in plants (Hammerstone et al. 2000; Joe et al. 2001). Evidences from both in vitro and in vivo studies have demonstrated that AP and purified procyanidine exhibit various biological functions such as antioxidant activity (Pearson et al. 1999; Eberhardt et al. 2000; Lee et al. 2003), modulation of the immune function (Kanda et al. 1998), reduction in adipose tissue mass (Nakazato et al. 2006), and prevention of muscle injury (Nakazato et al. 2010).

Nakazato et al. (2007) and Mizunoya et al. (2015) evaluated the isometric tetanic torque of the rat ankle joint and showed improved muscle endurance in animals fed with 5% AP. Furthermore, Mizunoya et al. (2015) examined the composition of the myosin heavy chain (MyHC) isoforms and myoglobin content of the skeletal muscle and showed that the relative amount of MyHC IIb was significantly reduced along with an increase in MyHC IIx content in rats fed with 5% AP. These findings suggest that the fast‐to‐slow MyHC change occurred in AP‐fed rats. These authors also reported an increase in the myoglobin content of the skeletal muscle.

To better understand the biological effects of dietary AP on muscle endurance, we evaluated the capillary density, mitochondrial content, and mitochondrial protein dynamics of gastrocnemius. Furthermore, the MyHC composition was examined, as reported by Mizunoya et al. (2015).

## Materials and Methods

### Animals, experimental diets, and ethical approval

Twenty‐four male Wistar rats (9‐week old) were obtained from Japan Clea (Tokyo, Japan). Rats were maintained at 23 ± 1°C under a 12:12 light/dark cycle. All animals were fed with laboratory chow (CE7; Clea, Japan) for 1 week and subsequently divided into the following three groups with no difference in their body weights: (1) 5% AP group (*n* = 8), (2), 0.5% AP group (*n* = 8), and (3), the control group (*n* = 8). To prevent variation between groups, we segregated all the animals to maintain similar average body weights. Compositions of the diet of each group are listed in Table 1. AP were provided by Asahi Breweries Ltd (Tokyo, Japan). Animals were maintained on the diets for 4 weeks and the weight of each animal was recorded after every 2 days throughout the experimental period. After the experimental period, animals were anesthetized by inhalation (isoflurane inhalation solution, Pfizer, NY) and sacrificed. The serum, gastrocnemius muscle, retroperitoneal adipose tissue (rWAT), epididymal adipose tissue (eWAT), mesenteric adipose tissue (mWAT), inguinal adipose tissue (iWAT), and brown adipose tissue (BAT) were obtained. After measuring their weights, these tissues were immediately frozen in liquid nitrogen and stored at −80°C for biochemical and histochemical analyses.

**Table 1:** Composition of the diets for the control and apple polyphenol groups

		Diet (g (%))	
Ingredients	Control	0.5%AP	5%AP
Casein	14.0 (14.0)	14.0 (13.93)	14.0 (13.33)
Cornstarch	62.0692 (62.0692)	62.0692 (61.76040)	62.0692 (59.11352)
Sucrose	10.0 (10.0)	10.0 (9.95)	10.0 (9.52)
Soybean oil	4.0 (4.0)	4.0 (3.98)	4.0 (3.81)
Cellulose	5.0 (5.0)	5.0 (4.98)	5.0 (4.76)
Mineral mixture	3.5 (3.5)	3.5 (3.48)	3.5 (3.33)
Vitamin mixture	1.0 (1.0)	1.0 (1.00)	1.0 (0.95)
L‐cystine	0.18 (0.18)	0.18 (0.179)	0.18 (0.171)
Choline(bitartrate)	0.25 (0.25)	0.25 (0.249)	0.25 (0.238)
t‐butylhydroquinone	0.0008 (0.0008)	0.0008 (0.00080)	0.0008 (0.00076)
AP	0 (0)	0.5 (0.50)	5 (4.8)

AIN mineral mixture and AIN vitamin mixture were obtained from Oriental Yeast Co., Tokyo, Japan. Composition of the control diet was based on AIN93M, as reported by the American Institute of Nutrition. 0.5% AP and 5% AP diets were modified from the control diet. AP, apple polyphenols.

All experiments were approved by the Animal Experimental Committee of Nippon Sport Science University (No. 010‐A01). The authors have read, and all experiments complied with, the policies and regulations of the Fundamental Guidelines for Proper Conduct of Animal Experiments and Related Activities in Academic Research Institutions published by the Ministry of Education, Culture, Sports, Science and Technology, Japan.

### Sodium dodecyl sulfate polyacrylamide gel electrophoresis (SDS‐PAGE) of MyHC isoforms

The content of MyHC isoforms in the gastrocnemius muscle was analyzed using SDS‐PAGE, as described by Mizunoya et al. (2008) with some modifications. The frozen tissues were homogenized and the total protein concentrations were measured using the Lowry's method (DC Protein Assay Kit, Bio‐Rad, CA). The samples were diluted in a 2× sample buffer (100 mmol/L dithiothreitol, 4% w/v SDS, 0.16 mol/L Tris‐HCl [pH 6.8], 43% glycerol, and 0.2% w/v bromophenol blue) and distilled water at a final protein concentration of 20 ng/*μ*L in 1 × sample buffer. These samples were heated at 100°C for 3 min and loaded. Electrophoresis was performed at a constant voltage of 140 V for 22 h except for the first 40 min, during which the maximum current was limited to 10 mA to facilitate penetration through the stacking gel. The entire gel unit was placed in an incubator at 4°C. After SDS‐PAGE, gels were stained with a silver staining kit (Silver stain KANTO III; Kanto Chemical Co., Tokyo, Japan) and dried.

### Immunohistochemical analysis

To investigate the effect of AP feeding on the capillary density in the gastrocnemius muscle, cryosections (6‐*μ*m thick) were fixed with Zamboni fixing solution for 15 min at room temperature. After being washed with 0.1 mol/L phosphate buffer (PB), sections were treated with methanol for 10 min at −20°C. Blocking was performed with 0.1 mol/L PB supplemented with 5% goat serum and 1% Triton X‐100 for 1 h at room temperature, followed by an overnight incubation with a primary antibody at 4°C. The primary antibody used in this study was that against platelet endothelial cell adhesion molecule‐1 (PCAM‐1) (1:100; 557355; BD Pharmingen, NJ), a known capillary marker. Costaining for the intramuscular connective tissue was performed using anti‐laminin antibody (1:500; sc‐16590; Santa Cruz Biotechnology, TX). After being washed with 0.1 mol/L PB, a secondary antibody (Alexa Fluor 555 goat anti‐mouse; 1:500; Invitrogen, CA, or Alexa Fluor 488 chicken anti‐rabbit; 1:1000; Invitrogen) was applied and the sections were incubated for 1 h at room temperature. Sections were mounted using VECTASHIELD with 4′,6‐diamidino‐2‐phenylindole (DAPI; Vector Laboratories, England) and the signals were observed using a fluorescence microscope (Olympus BX 60, Olympus, Tokyo, Japan) equipped with a digital color camera. Photographs were obtained for the superficial part and deep part of the gastrocnemius muscle, and the number of signals in 1 mm^2^ (superficial part 0.5 mm^2^; deep part 0.5 mm^2^) area was counted per sample using ImageJ software.

### Western blot analysis

Western blot analysis was performed using the gastrocnemius muscle to investigate if AP feeding affects mitochondrial oxidation ability and biosynthesis of molecules involved in angiogenesis. We examined the expression levels of peroxisome proliferator‐activated receptor gamma coactivator 1‐alpha (PGC‐1α), electron transport system enzyme complex I–V, dynamin‐related protein 1 (Drp1), mitofusin 2 (Mfn2), and optic atrophy 1 (Opa1). Furthermore, the expressions of thrombosponsin‐1 (TSP‐1) and vascular endothelial growth factor (VEGF) as factors related to angiogenesis were examined. The frozen tissues were homogenized using radioimmunoprecipitation assay (RIPA) buffer (Thermo Fisher Scientific, MA) containing protease inhibitors (Complete Mini, Roche Diagnostics, Basel, Switzerland), and the total protein concentrations were measured using the Lowry's method (DC Protein Assay Kit, Bio‐Rad). Equal amounts (10–20 *μ*g) of proteins were separated by SDS‐PAGE and transferred onto a nitrocellulose membrane or polyvinylidene fluoride (PVDF) membrane. The transferred membranes were blocked with 5% (w/v) fat‐free milk in Tris‐buffered saline containing 0.1% (v/v) Tween‐20 (TBST) at room temperature for 1 h with gentle shaking and overnight incubated with primary antibodies at 4°C.

The primary antibodies used in this study were as follows: Total OXPHOS rodent WB antibody cocktail (1:5000; ab 110413; Abcam, Cambrige, UK), Drp1 rabbit mAb (1:1000; #5391; Cell Signaling Technology, Danvers, MA), Mfn2 rabbit mAb (1:1000; #9482; Cell Signaling Technology), purified mouse anti‐OPA1 (1:500; 12606; Biosciences Inc., Allentown, PA), anti‐PGC‐1 (1:1000; AB 3242; Millipore, Darmstadt, Germany), anti‐TSP‐1 (1:500; sc‐59886; Santa Cruz Biotechnology), and anti‐VEGF (1:500; sc‐507; Santa Cruz Biotechnology).

After being washed with TBST, the membranes were incubated at room temperature for 1 h with horseradish peroxidase‐labeled anti‐rabbit or anti‐mouse immunoglobulin (Cell Signaling Technology) as the secondary antibody. The blots were washed with TBST and proteins were detected with chemiluminescent reagents (Super Signal West Pico Chemiluminescent Substrate, Thermo Fisher Scientific), followed by visualization with a CCD imager (ChemiDoc XRS Plus, Bio‐Rad) and quantification using Image Lab software. Ponceau S staining (Po‐S; SERVA) was used for a loading normalization.

### Real‐time polymerase chain reaction (PCR)

The mRNA level of TSP‐1 was evaluated using reverse transcriptase and real‐time PCR (RT‐PCR). Total RNA was extracted (TRIzol Reagent; Invitrogen) from the gastrocnemius according to the manufacturer's instructions, and the total RNA concentration and purity were measured by spectrophotometry. cDNA synthesis from total RNA (1.5 *μ*L) was performed using High Capacity cDNA Reverse Transcription kit (Thermo Fisher Scientific). After cDNA synthesis, real‐time quantitative PCR amplification was performed by THUNDERBIRD SYBR qPCR Mix kit (Toyobo, Osaka, Japan). The amplification was performed with a thermal cycler (CFX96 Real‐Time System; Bio‐Rad) using the following program: 30 sec at 95°C, 5 sec at 95°C, and 60 sec at 60°C for 40 cycles. External standard curves were used for the quantitative analysis, and the mRNA level of the target gene was normalized to that of *β*‐actin, the housekeeping control gene. We confirmed that the critical threshold (Ct) value of *β*‐actin was similar among the three groups. The sequences of the primers used in this study were as follows: 5′‐GGAAGAGCATCACGCTGTTTG‐3′ (forward primer) and 5′‐GCGCTCTCCATCTTGTCACA‐3′ (reverse primer) for TSP‐1, 5′‐TTCAACACCCCAGCCATGT‐3′ (forward primer) and 5′‐GTGGTACGACCAGAGGCATACA‐3′ (reverse primer) for *β*‐actin. A comparative Ct method was applied using standard curves, with the amount of target gene normalized to that of the endogenous control gene.

### Statistical analysis

All the values are presented as the mean ± standard error (SE). To compare the mean among three groups, one‐way analysis of variance (ANOVA) and Tukey–Kramer posthoc test were performed. Statistical significance was defined as *P* < 0.05, and the trend was defined as *P* < 0.10. Statistical analyses were performed on a Windows computer using a statistical software package (SPSS Japan, Inc., Japan).

## Results

### Body weight, food intake, and tissue weight

**Figure 1:**
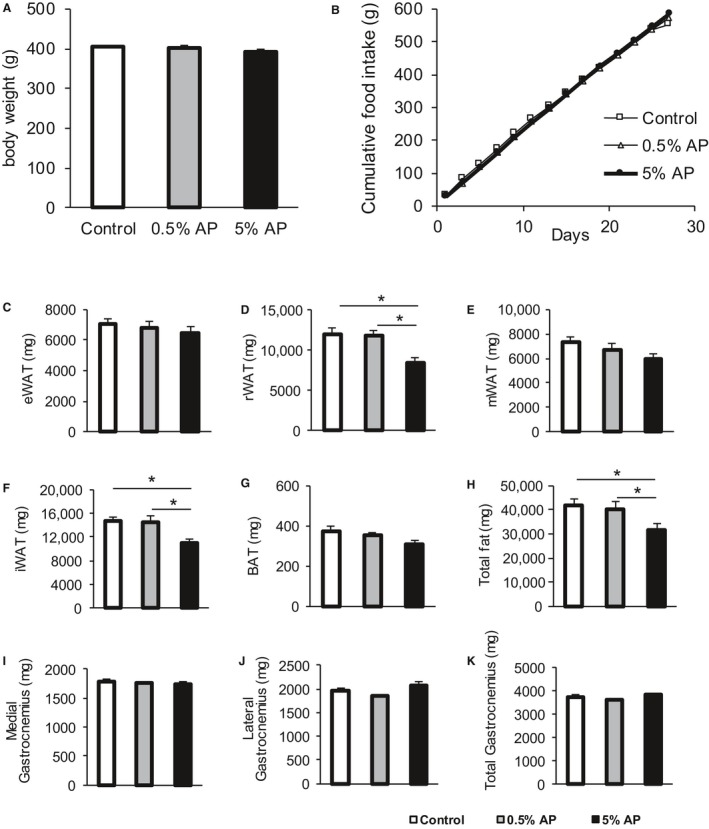
Cumulative food intake, body weight, and tissue weight. Effect of dietary AP on (A) the body weight (B) cumulative food intake, (C–H) adipose tissue weights, and (I–K) gastrocnemius muscle weight. The open bar represents the control group, the shaded bar represents the 0.5% AP group, and the closed bar represents the 5% AP group. Data are represented as mean ± SE. Statistical significance was evaluated by one‐way ANOVA with Tukey–Kramer posthoc test. Statistical significance was expressed by *P < 0.05. AP, apple polyphenols.

To investigate the influence of AP feeding, the body weights, amount of food consumed, and tissue weights were measured. The average body weight of the group fed with 5% AP, 0.5% AP, Control was 392.9 ± 5.8 g, 401.0 ± 7.3, 403.9 ± 5.4 g, respectively. There was no significant difference among these three groups (Fig. 1A).

The measurement of the weight of the adipose tissue showed no significant difference between eWAT and mWAT; however, the weight of rWAT and iWAT significantly decreased. The total weight of the excised adipose tissue was significantly lower in the group fed with 5% AP than the control group and the group fed with 0.5% AP. In addition, the decrease in the adipose tissue weight was dependent on the dosage of AP (Fig. 1C–H). The evaluation of the muscle weight of the medial and lateral gastrocnemius muscle revealed no significant difference between the control, 0.5% AP, and 5% AP groups (Fig. 1I–K).

### Isoforms of MyHC

**Figure 2:**
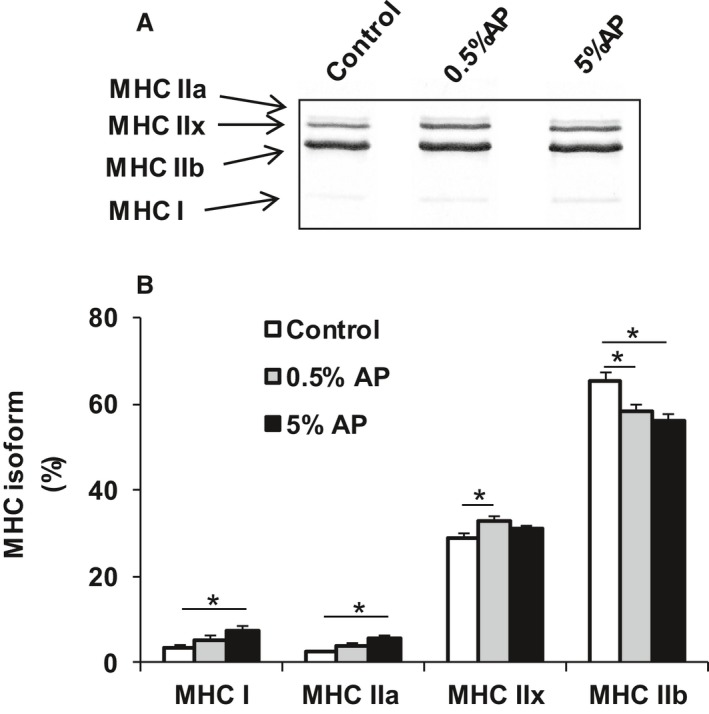
MyHC ratio in the gastrocnemius following AP treatment. Gastrocnemius muscles from the control, 0.5% AP, and 5% AP diet groups were analyzed for the ratio of each MyHC isoform (%) relative to total MyHC. (A) Representative SDS‐PAGE. (B) Densitometry. The open bar represents the control group, the shaded bar represents the 0.5% AP group, and the closed bar represents the 5% AP group. Data are represented as mean ± SE. Statistical significance was evaluated by one‐way ANOVA with Tukey–Kramer posthoc test. Statistical significance was expressed by *P < 0.05. AP, apple polyphenols.

We evaluated the composition of the fiber by SDS‐PAGE to investigate if AP exert effects on the muscle endurance through variations in MyHC isoforms (MyHC type I, IIa, IIx, and IIb expressed in the order of the slowest to the fastest). Total myofibers were considered as 100% and the proportion of each MyHC was evaluated. MHC IIb was significantly lower in the group fed with 0.5% AP (58.1 ± 1.8%) and 5% AP (55.9 ± 1.5%) than in the control group (65.1 ± 2.0%). On the other hand, the percentage of MyHC I was significantly higher in the group fed with 5% AP (7.6 ± 1.0%) than in the control group (3.3 ± 0.9%) (Fig. 2). Furthermore, the variation in the MyHC isoforms following oral intake of AP dependent on the dosage of AP.

### Capillary density and angiogenesis

**Figure 3:**
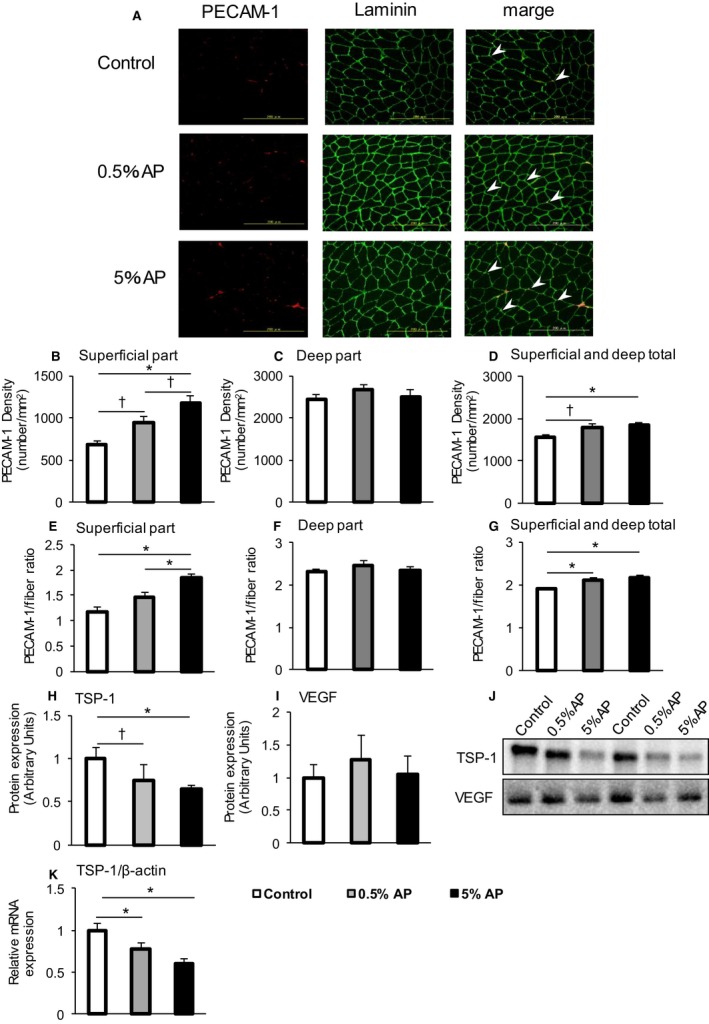
Measurements of the capillary density of the gastrocnemius muscle. (A) Representative platelet endothelial cell adhesion molecule‐1 (PCAM‐1, left panel), laminin (central panel), and merge (right panel) staining of medial gastrocnemius muscle. Control (upper panel), 0.5% AP (middle panel), and 5% AP (bottom panel) are shown. Arrow heads indicate representative PCAM‐1 signal. (B–D) PCAM‐1 density is shown (superficial part, deep part, superficial and deep total, respectively). (E–G) PCAM‐1/fiber ratio is shown (superficial part, deep part, superficial and deep total, respectively). (H, I) TSP‐1 and VEGF protein levels. (J) Representative western blot. (K) TSP‐1 mRNA expression. The open bar represents the control group, the shaded bar represents the 0.5% AP group, and the closed bar represents the 5% AP group. Data are represented as mean ± SE. Statistical significance was evaluated by one‐way ANOVA with Tukey–Kramer posthoc test. Statistical significance was expressed by *P < 0.05 and ^†^ *P* < 0.1. AP, apple polyphenols

To investigate the mechanism underlying the improvement in the muscle endurance following dietary administration of AP, the capillary density was evaluated. The density of the capillary vessels was determined by immunohistochemical staining. The results showed 70% higher staining in the superficial part of the gastrocnemius muscle from the group fed with 5% AP than the control group (Fig. 3B). However, no significant difference was found between the staining intensity in the deep part between the three groups (Fig. 3C). The total signal from the superficial and deep parts from the group fed with 5% AP was 17.8% higher than that from the control group (Fig. 3D). The fiber ratio in the superficial part was increased by 58% in the group fed with 5% AP than the control group, and a similar trend in the capillary density was observed (Fig. 3E).

We evaluated the expression of factors that cause an increase in the blood vessel density. The expressions of TSP‐1 and VEGF proteins known to be involved in angiogenesis in the gastrocnemius muscle were examined by western blot analysis. In comparison with the control group, those fed with 0.5% and 5% AP showed a 25% and 35% decrease in TSP‐1 protein expression, respectively (Fig. 3H). No significant change in VEGF expression was observed (Fig. 3I).

The protein TSP‐1, one of the matricellular glycoproteins, is a circulating angiostatic protein (Iruela‐Arispe et al. 1991; Kong et al. 2014). The expression of TSP‐1 has been reported to be high in the adipose tissue. In this study, we examined TSP‐1 mRNA expression in the muscle and TSP‐1 protein expression in the adipose tissue to determine if TSP‐1 is produced by muscles cells in the gastrocnemius or externally supplied from the adipose tissue via circulation.

**Figure 4:**
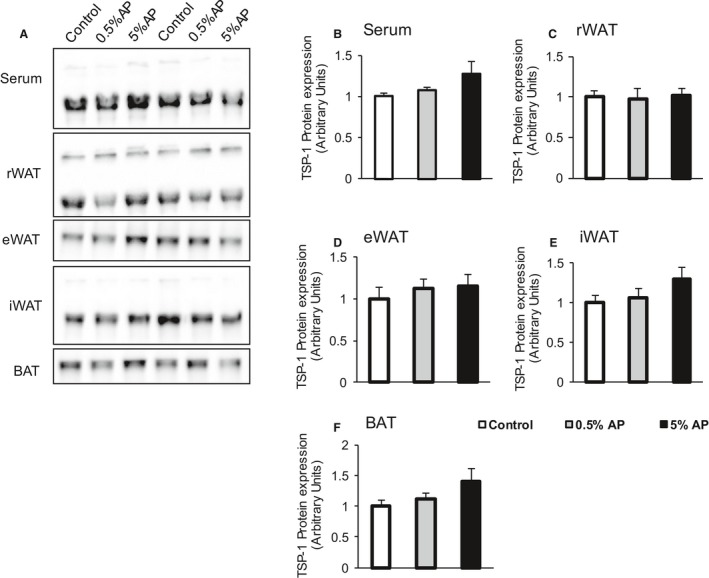
Effect of dietary AP on the expression level of thrombosponsin‐1 (TSP‐1). (A) Representative western blot. (B‐F) TSP‐1 protein level in the serum, retroperitoneal adipose tissue (rWAT), epididymal adipose tissue (eWAT), inguinal adipose tissue (iWAT), and brown adipose tissue (BAT). The open bar represents the control group, the shaded bar represents the 0.5% AP group, and the closed bar represents the 5% AP group. Data are represented as mean ± SE. Statistical significance was evaluated by one‐way ANOVA with Tukey–Kramer posthoc test. AP,apple polyphenols.

The results of the expression analysis of TSP‐1 protein in the adipose tissues were similar among groups (Fig. 4). On the contrary, the mRNA levels significantly decreased by 26% and 41% in groups fed with 0.5% and 5% AP, respectively, as compared to the control group (Fig. 3K).

### Mitochondrial content and dynamics

**Figure 5:**
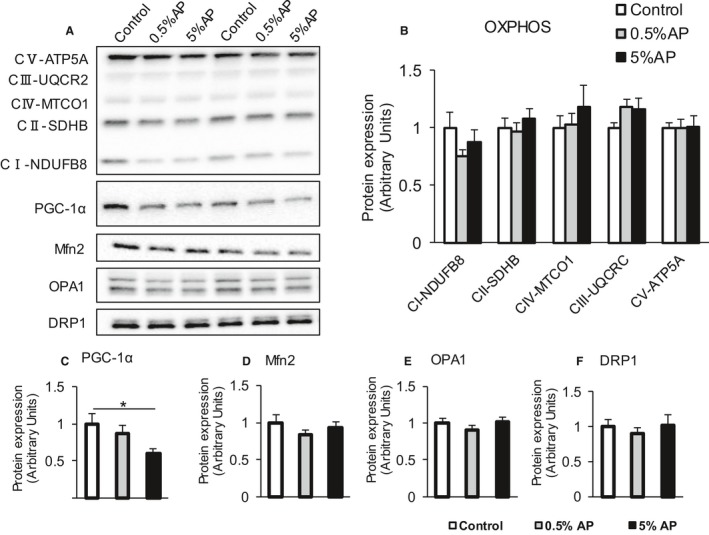
Effect of dietary AP on the mitochondrial function in gastrocnemius. Representative western blot. (B–F) Protein levels of OXPHOS, PGC‐1α, OPA1, Mfn2, and Drp1, respectively. The open bar represents the control group, the shaded bar represents the 0.5% AP group, and the closed bar represents the 5% AP group. Data are represented as mean ± SE. Statistical significance was evaluated by one‐way ANOVA with Tukey–Kramer posthoc test. Statistical significance was expressed by *P < 0.05. AP,apple polyphenols.

To elucidate the molecular mechanism underlying the improvement in the muscle endurance induced by the dietary administration of AP, the mitochondrial contents were examined as the total content of five different electron transport chain (ETC) complex subunits by western blot analysis. No significant difference was observed between the groups in the levels of all complexes I–V (Fig. 5B). PGC‐1α is a transcriptional coactivator that regulates metabolism in numerous tissues and thought to play a central role in the mitochondrial biosynthesis (Wu et al. 1999; Russell et al. 2004). The level of PGC‐1α protein in the gastrocnemius was lower in the 5% AP group than the control group (Fig. 5C).

The proteins involved in the mitochondrial fusion–fission dynamics play a crucial role in many cell processes, and these dynamics control mitochondrial morphology, which in turn influences several important mitochondrial properties such as the mitochondrial bioenergetics and quality control (Karbowski and Youle 2003; Ishihara et al. 2006; Frölich et al. 2013). To investigate the effect of AP administration on the mitochondria dynamics in gastrocnemius, levels of Mfn2 and Opa1 proteins involved in fusion were evaluated. In addition, the expression of Drp1 protein involved in fission was evaluated. As a result, no significant differences were observed in the expression levels of all these proteins between three groups (Fig. 5D–F).

## Discussion

In this study, we investigated the mechanism underlying the improvement in the muscle endurance following dietary intake of AP in rats. We confirmed that the adipose tissue mass in rats treated with AP was significantly lower than that in rats from the control group, consistent with the results of a previous report (Nakazato et al. 2006). In addition, the relative level of MyHC IIb was significantly reduced along with the compensatory increase in MyHC I and MyHC IIa levels in the group fed with 5% AP, similar to that observed by Mizunoya et al. (2015). In this study, we observed that the capillary density in gastrocnemius increased in AP‐treated gastrocnemius. Furthermore, the content of TSP‐1, the negative regulator of angiogenesis, was significantly lower in the treatment group than the untreated control.

The most important finding of this study is the higher capillary density observed in AP‐treated animals. Induction of high vascularity after AP treatment was apparent in the superficial region of gastrocnemius. In the gastrocnemius muscle, slow muscle fibers were abundant in the deep region. High capillary density was also observed in the deep region of gastrocnemius. These results are in line with those previously reported showing that capillaries are abundant around slow muscle fibers (Ripoll et al. 1979; Browning et al. 1996; Staron et al. 1999). The deep region of gastrocnemius may exhibit the slow muscle phenotype and show no effect of AP treatment. On the other hand, the fast fibers in the superficial part acquired the property of slow muscle following AP treatment.

We investigated the molecular mechanisms underlying the increase in the capillary density following AP treatment. VEGF and TSP‐1 are well known regulators of angiogenesis. In the present study, we found that the expression of TSP‐1 was significantly lower in AP‐treated gastrocnemius than the control muscle. TSP‐1 is a large extracellular matrix glycoprotein known to aggregate platelets (Mosher 1990), inhibit endothelial cell proliferation (Bagavandoss and Wilks 1990), prevent cell spreading (Lawler et al. 1988), facilitate wound healing (DiPietro et al. 1996), disrupt focal (cell‐to‐matrix) adhesions (Murphy‐Ullrich and Höök 1989), and inhibit angiogenesis in vitro and in vivo (Rastinejad et al. 1989; Iruela‐Arispe et al. 1996). Audet et al. (2013) reported that the chronic exposure to a TSP‐1 mimetic (ABT‐510) resulted in a decrease in the skeletal muscle capillarity. Malek and Olfert (2009) also reported that the global deletion of TSP‐1 resulted in an increase in the cardiac and skeletal muscle capillarity. We observed a decrease in TSP‐1 mRNA level in AP‐treated gastrocnemius, suggesting that TSP‐1 expression in the skeletal muscle was repressed by AP treatment. Taken together, we conclude that AP treatment decreased TSP‐1 translation and consequently TSP‐1 content in the skeletal muscle.

The protein TSP‐1 is produced by a wide variety of cells and affects multiple organs through blood circulation (Kong et al. 2014). TSP‐1 is highly expressed in visceral adipose tissues, and its expression is elevated during obesity and inflammation of the adipose tissue in rats and humans (Hida et al. 2000; Varma et al. 2008). Thus, we analyzed TSP‐1 protein level in the adipose tissue. However, no significant difference in TSP‐1 level was observed in the adipose tissues from AP‐treated groups and control group. Therefore, we suggested that the decrease in TSP‐1 protein expression in the gastrocnemius muscle was caused by the decrease in its mRNA expression in the skeletal muscle.

The content and quality of mitochondria is important in determining the endurance capacity. In this study, we found that the contents of the mitochondria and the fusion/fission‐related proteins were unaffected following AP treatment. PGC‐1α is an important key regulator of energy metabolism and activates mitochondrial biogenesis in the skeletal and cardiac muscles (Lin et al. 2002; Russell et al. 2004). In the present study, PGC‐1α protein level in the gastrocnemius was decreased by AP treatment. Thus, the improvement in the muscle endurance following the oral intake of AP may not be associated with the mitochondrial amount and quality. The overexpression of PGC‐1α in the skeletal muscle induced high capillary density (Arany et al. 2008; Chinsomboon et al. 2009; Patten et al. 2012). On the other hand, the elevated expression of PGC‐1α in the vascular endothelial cells inhibited angiogenesis (Sawada et al. 2014). Thus, PGC‐1α may exhibit opposite functions such as promotion of angiogenesis in the muscle and angiogenesis inhibition in the vascular endothelial cells. Whether the attenuation in the expression of PGC‐1α was associated with the muscle or vascular endothelium is questionable.

Both MyHC shift and capillary density increase were induced by AP treatment. As mentioned above, Malek and Olfert (2009) also reported that the global deletion of TSP‐1 increases the skeletal muscle capillarity. These authors observed high endurance capacity in TSP‐1‐deleted mice. No significant differences in the fiber composition and oxidative enzyme activity were observed in TSP‐1 null mice as compared to the wild‐type mice. The increase in the capillary density has significant effects on the enhancement in the endurance capacity.

To analyze the detail mechanisms underlying the in vivo effect of AP intake, it is necessary to identify the compound directly involved in the process. It is uncertain whether the same compound induced changes in MyHC compositions and capillary density. There is also a possibility that multiple components act in a synergistic manner. Procyanidin is the most abundant component of AP that may be a possible candidate. Other components such as phenolic acids and dihydrochalcones are promising candidates. It is thought that it can be clarified by investigating the influence when orally intake the polyphenol from which these ingredients were fractionated.

In summary, we conclude that the AP treatment induced high capillary densities in the rat skeletal muscles. TSP‐1 decrease played a key role in this phenomenon. Dietary AP may have no effect on the amount and quality of mitochondria. Although the details of the mechanisms are still uncertain, AP have the potential of improving the skeletal muscle performance. As dietary AP also strongly inhibited adipogenesis, AP treatment may have therapeutic applications for type II diabetes and cardiovascular disorders.

## Conflicts of Interest

Research materials and grant were provided by Asahi Breweries Ltd. However, the sponsor had no control over the study design, experiments, interpretation, writing, or publication of this work.
